# Ethics in extracorporeal life support: a narrative review

**DOI:** 10.1186/s13054-021-03689-0

**Published:** 2021-07-21

**Authors:** Alexandra Schou, Jesper Mølgaard, Lars Willy Andersen, Søren Holm, Marc Sørensen

**Affiliations:** 1 Department of Anaesthesiology and Intensive Care Medicine, Aalborg University Hospital, Hobrovej 18-22, 9100 Aalborg, Denmark; 2 Centre for Cancer and Organ Diseases, Rigshospitalet, Copenhagen University Hospital, Blegdamsvej 9, 2100 Copenhagen Ø, Denmark; 3 Heart Centre, Rigshospitalet, Copenhagen University Hospital, Blegdamsvej 9, 2100 Copenhagen Ø, Denmark; 4 Department of Law, School of Social Sciences, Faculty of Humanities, University of Manchester, Williamson Building, Oxford Road, Manchester, M13 9PL UK

**Keywords:** Extracorporeal life support, Extracorporeal membrane oxygenation, Ethics

## Abstract

During 50 years of extracorporeal life support (ECLS), this highly invasive technology has left a considerable imprint on modern medicine, and it still confronts researchers, clinicians and policymakers with multifarious ethical challenges. After half a century of academic discussion about the ethics of ECLS, it seems appropriate to review the state of the argument and the trends in it. Through a comprehensive literature search on PubMed, we identified three ethical discourses: (1) trials and evidence accompanying the use of ECLS, (2) ECLS allocation, decision-making and limiting care, and (3) death on ECLS and ECLS in organ donation. All included articles were carefully reviewed, arguments extracted and grouped into the three discourses. This article provides a narrative synthesis of these arguments, evaluates the opportunities for mediation and substantiates the necessity of a shared decision-making approach at the limits of medical care.

**Supplementary Information:**

The online version contains supplementary material available at 10.1186/s13054-021-03689-0.

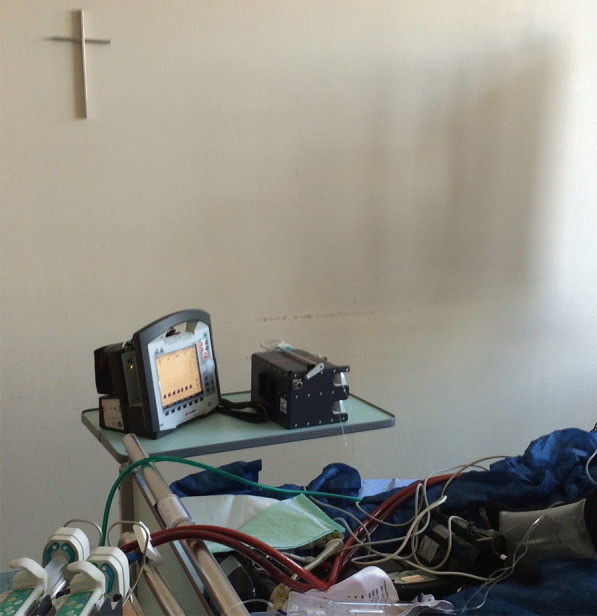

## Background

### The state of extracorporeal life support

Since the beginning of extracorporeal life support (ECLS)^1^ in the early 1970s the treatment has changed significantly [1, 2]. The technique^2^ has been miniaturised and made more durable and biocompatible. Most importantly, ECLS has evolved from a risky experiment to a standard treatment option for critical respiratory or cardiac failure. Expanding knowledge, centralised care and, not least, the support of a well-established international consortium entrusted with data registry and the development of clinical standards have made ECLS a feasible procedure, serving a growing number of increasingly complex and diverse patients.

Nonetheless, as the social costs of ECLS remain high its appropriateness is frequently questioned. However, the ethical concerns mainly focus on specific aspects. To date, none of the numerous, exponentially proliferating publications on the ethics of ECLS, have covered the whole spectrum of moral implications.

## Method

For the purpose of a broad orienting overview on the existing ethical debate, a comprehensive search was carried out in PubMed on 31 October 2020 under the search terms: “ECLS” OR “extracorporeal life support” OR “ECMO” OR “extracorporeal membrane oxygenation” AND “ethics”.

This returned 380 titles of which 236 articles were excluded upon inspection of title and abstract. One hundred and ninety-seven articles were not dealing with an ethical subject; 27 were not electronically accessible; nine were written in a language other than English; and three were re-publications. All of the remaining 144 articles were thoroughly categorised according to their format and provenance, classed with an ethical discourse and summarised with regard to their ethical content (see the interactive Additional file 1 online).

Based on this research we identified three ethical discourses, each covering several closely connected issues: (1) the research-ethical discourse on trials and evidence of ECLS (a summary of contributions on the ethical impact of the knowledge about ECLS and its collection); (2) the clinical and public policy ethical discourse on ECLS allocation, decision-making and limiting care (a summary of contributions on the ethical impact of ECLS on the omission and withdrawal of therapy); and (3) the clinical and public policy ethical discourse concerning death on ECLS and extracorporeal interval support for organ retrieval (EISOR)^3^ (a summary of contributions on the ethical impact of ECLS on the definition of death and organ donation). The following text was written in three chapters as a narrative synthesis of the most prominent arguments. More recent articles were selectively included.

## Aims and scope

Although ECLS covers different types of support, different indications and goals as well as patients of very different ages and clinical conditions, all conceivable scenarios of ECLS seem to be fraught with high economic and personal costs, more or less limited evidence and, most often, a lack of promising alternatives. None of the most central of the ethical issues mentioned (What evidence should we demand for treatment? When should the treatment be withheld or withdrawn? When should it be allowed to harvest organs from donors?) are necessarily different from the ethical issues in the use of life support systems in general. Rather, the uniqueness of ECLS lies in the simultaneity of these issues, their complexity and their acuteness and aggravation due to a rapidly growing clinical demand. The aim of this review is to unfold the current state of the discourses, to integrate and evaluate them.

ECLS in the Coronavirus disease 2019 (COVID-19) pandemic and in paediatrics are included in the sections, as their ethical issues are to a great extent subordinated to the aforementioned discourses. For example, the rationing of ECLS under COVID-19 may be subordinated to the allocation of ECLS in general, while the deliberative role of parents of infants on ECLS may be subordinated to the ethical discourse of (subsidiary) decision-making. Where relevant, however, the sections will deal explicitly with the normative differences between children and adults, and the exceptional role of the pandemic.

## The ethical impact of trials and evidence on ECLS

### Trials

Gaining knowledge about the impact of ECLS on mortality has always faced researchers with complex ethical dilemmas. The development of evidence underlying ECLS is irrevocably intertwined with the design of the trials that generate that evidence, which therefore remains limited and largely retrospective. There are currently eight randomised controlled trials on the subject, six of which deal with respiratory failure [3, 4]. The two trials on neonates show significant advantage from ECLS, whereas only one out of four trials on respiratory failure in adults corroborates this outcome. Regarding cardiac failure, a minor prospective single-centre study from 2019 does not indicate any significant benefit from V-A ECMO in cardiogenic shock [5]. In extracorporeal cardiopulmonary resuscitation (ECPR), in contrast, a recent randomised controlled trial displays improved survival from out-of-hospital cardiac arrest (OHCA) with favourable neurological performance in the ECLS-group [6].

As the only overlap of all of these studies, ECLS was applied as an immediate, life-saving bridge to spontaneous recovery or restoration. The underlying patient pool is therefore extremely heterogeneous, and the favourable outcomes for children, especially neonates, are commonly attributed to their particular stage of maturation or effective surgery. Prospective research indicates that the benefits of ECLS for adult patients are far more questionable.

Correspondingly, the evidence for ECLS and its ethical value have been discussed from the beginning. Even before publication of the first randomised controlled trial in 1979, the evidence-related lack of alternatives to randomisation was both proclaimed and contested in the same journal issue [7]. Chalmers and Miké et al. consider randomisation the gold standard for clinical research, and as such represent a distinctive standpoint among early studies [8, 9]. Conversely, Lantos et al. highlight the reductionism associated with well-designed randomised trials, which were necessarily preceded by a certain degree of practical knowledge and expectation, ethical values and inherent uncertainty. Randomising extremely vulnerable patients to a given trial’s control treatment, which the investigator believes to be inferior, seemed unacceptable [10]. Twenty-five years later, the investigators of the EOLIA trial on the use of ECLS in the acute respiratory distress syndrome (ARDS) would be confronted with the same dilemma, and opted to terminate the study [4].

Following the initial discussion, Bartlett et al. and O’Rourke et al. applied different adaptive study designs in their neonatal trials in the 1980s [11]. These approaches were later criticised for only minimising rather than solving the major ethical problem of randomisation [3]. In his influential article from 1992, Truog identified some ECLS-specific features that limit the role and feasibility of randomisation: the rapid and ongoing development of clinical standards and the unacknowledged need for large-scale demographic data and long-term follow-up [12]. Recently, Bartlett and Gattinoni et al. have revisited this viewpoint [3, 13]. In a later editorial, Truog outlines the limitations of obtaining the intensive care patient’s informed consent to inclusion in clinical trials [14]. For this reason, in the adaptive studies, consent was only sought from parents whose children had already been randomised to the intervention group.

Worrall argues that the ethics of randomisation in ECLS research are conflated with the underlying concept of evidence. Indeed, he shows that the clear and robust concept of evidence is anything but a given. Different interpretations yielded different ethical conclusions [15].

### Evidence

In a response to Lantos et al. from 1993 Miké et al. introduced what they call the concept of ethics of evidence, which brings together the professionals’ moral duty to contribute to the best statistical evidence about ECLS and to a deliberative assessment of the remaining uncertainty in a multilateral process involving both patients and society [9]. On this reading, it should be fairly uncontroversial.

However, Miké et al. overlook that evidence is not the starting point of deliberation, rather its product, at a specific point of time during a reflective communication process. Formal, prospective research, as Lantos et al. put it, is always preceded by some sort of more or less informal historical experience, as the design of trials presupposes expectation and deselection [10]. Both the use of an unproven treatment with clinical equipoise and randomisation without equipoise can be equally unethical. Ultimately, the review and publication process is not merely follow-up, but an essential, integral element of research itself.

Miké et al. would therefore benefit from incorporating Lantos’ moral scruples, since ethics apply from the beginning of an inquiry, not only at the end [16]. Both clinical and scientific evolution entail collective learning. This is a unidirectional movement, in which the agents involved irrevocably adopt a new mental state—the time after ECLS can never again be the time before ECLS. In this process, randomised trials are superior to other studies when it comes to detecting the impact of an unproven treatment, but are inferior to other study designs at answering the question of *how* this impact is working—let alone what these findings *mean* in their clinical context.

## Resource allocation, decision-making and limiting care on ECLS

### Resource allocation

Appropriate ECLS-utilisation is extensively discussed due to its heavy staffing and logistical requirements. While several historical cost-effectiveness analyses have shown it to be cost-efficient for some patient populations and indications in certain national health systems, at least compared to other highly cost-intensive interventions, this is not universally the case [1]. Initiating or continuing ECLS indiscriminately conflicts at a minimum with physicians’ duty to allocate resources responsibly [17, 18]. However, the lack of clear evidence limits the use of prospective economic calculations. Allocation decisions surrounding the use of ECLS are already made urgently with a lack of time, information and therapeutic alternatives. The introduction of ECPR in cardiac arrest has made these decisions even more time sensitive. Brauner et al. hold that similar economic forces that led to CPR being indicated in any event of cardiac arrest were currently driving the expansion of indications for ECLS [19]. Understanding these forces would be essential for establishing a more appropriate practice.

As more evidence regarding the impact of ECLS emerges, the calculation of cost per life-year saved [1, 20] and regular cost–benefit analyses [18] might assist decision-making. Quality-adjusted life-years might be part of this evaluation, yet they would contain the patient’s subjective judgements [20] and would not consider the decision’s societal impact [21]. In healthcare systems based on private health insurance, resource allocation might depend on the payer’s willingness to cover the costs, which could result in unequal access to ECLS [1]. To ensure that the most vulnerable patients are protected, in times of scarcity ECLS resources should be prioritised at a national health policy level, guided by pre-existing rationing plans [20, 22–24].

The COVID-19 pandemic has further focused attention on prioritisation [25, 26]. Rather than adding novel ethical conflicts, ECLS ultimately changed state from being relatively limited to absolutely scarce. Tyrrell et al. provide a systematic overview of different national recommendations referring to the traditional concepts of utilitarianism and egalitarianism [27]. Those might be still reconcilable if utility is reframed as beneficence and equality as equity [28]. DeLaney et al. argue for frequently reviewing disaster-protocols aimed at assessing ECLS-prioritisation that takes into account the individual’s societal and instrumental value, as well as the primacy of the youngest patients and those with the longest life expectancy [29]. However, while few authors would disagree that those in the greatest need should be prioritised during an ECLS-shortage, they may dissent on what constitutes “the greatest need”: the prospect of survival, when the alternative is death? Survival for a full life cycle? How to weight the likely survival of one individual against the less likely survival of many? Should an older patient with good recovery prospects but a short remaining lifespan give way to two children with unpredictable or poorer outcomes [30]?

### Decision-making

Most authors emphasise that the key to a successful ECLS course would be clear and timely communication, ensuring that the individual’s perspective is included in the decision to initiate or (dis-)continue therapy [31]. In elective cases of expected (post-procedural) ECLS, an informed consent should be obtained in advance [32].

Shared decision-making may enhance patient autonomy in awake patients, but it also presupposes the patient’s cognitive capacity to process the information, and the patient or surrogates may find the burden of weighty decisions overwhelming. Misunderstanding, emotional distortion, patients’ trust in expertise, urgency and lack of prognostic certainty make true informed consent difficult to achieve and basic ethical principles hard to apply [20, 31, 33]. Consent would often be implied, but rarely explicitly sought before initiating ECLS [32]. Moreover, true consent for *de*cannulation could not be properly achieved before cannulation, as consent might be given under duress [18]. Perhaps for these reasons, the majority of physicians counter-intuitively advocated for their discretion in limiting V-A ECMO in a notable survey conducted by Meltzer et al. in 2016 [35]. Three years later, Abrams et al. confirmed this trend with regard to V-V ECMO [36]. Whitman and Bein et al. congruently focus on the limits of patient and surrogate responsibility and the professionals’ obligation for counselling and guidance [31, 37, 38]. Given a lack of cognitive capacity, those acting on behalf of patients should always act in the patient’s best interest [21].

### Limiting care

As the objective of ECLS is to help patients survive until recovery or restitution, initiating or continuing ECLS is generally considered inappropriate if there are no such prospects [39]. This applies both to pre-ECLS patients and to those already undergoing it, as most authors suggest that withholding and withdrawing ECLS are ethically equivalent [17, 38, 40, 41]. However, some highlight the cruelty of withdrawing ECLS from objecting patients [17, 18]. For patients trapped on this metaphorical bridge to nowhere, a no-escalation-strategy might provide a way out [42]. DeMartino et al. claim that ECLS should not be withheld because withdrawal seems unethical. To do so would deny patients an opportunity while their prognosis is clarified [34].

Paris et al. state that the concept of professional responsibility should be understood in a principlistic way [43]. The quality of life principle should be integrated with the sanctity of life principle of personalism [21, 23, 44]. Meanwhile, the concept of futility is generally regarded as flawed if the deliberation does not take account of the patient’s wishes and expectations [45]. The pivotal point of the ethical debate is the extent of this deliberation—i.e. whether professional integrity and responsibility on the one hand or patient autonomy on the other hand should be the decisive factor in the event of disagreement. Preemptive ethics consultation, daily interdisciplinary rounds, and early advance care planning that addresses values, appropriate goals and fears, as well as support from spiritual and palliative care providers, are widely recommended [20, 35, 38, 46].

## Defining death on ECLS and the use of EISOR

### Death on ECLS

It is difficult to find an operational definition of human life, and the same is true of death. As circulation is halted, and organs are deprived of oxygen, they start dying off at different speeds. Further, the invention of the ventilator and extracorporeal circulation has forced a new thinking, inasmuch as the loss of respiration and heart function no longer mean death and brain function was made central in the concept of human life [47]. According to Baker et al. consciousness and self-awareness were the only emergent phenomena whose loss would be at once necessary and sufficient to define the death of a person [48]. As advancements in intensive care and organ transplantation necessitated a more reproducible diagnostic tool for the determination of death, in 1968 the brain death criteria were established, which defined the clinical signs of the irreversible loss of vital brain function [49].

In order to maximise the organ yield, in 1981 Bernat adopted a prospective death criterion based on the concept of permanence, where “irreversible” denoted a condition that, like in brain death, *could not* be reverted by any known technologies, “permanent” denoted a condition that *would not* be reverted, and therefore become irreversible [48]. Bernat’s concept of the permanence of death implies that death is technologically dependent, because the exact moment at which the cessation of brain activity changes from permanent to irreversible can be modified with ECLS—and, in the absence of international standards, it might also differ from place to place [50]. In an early contribution on EISOR, Shemie holds that critics of this dependence typically fail to recognise that the concept of death would have inevitably and always (independently from ECLS) been definitional rather than clearly descriptive [47]. Verhejde et al. argue that not even permanence could be empirically determined, since there were no reliable tests to confirm this state [51]. However, Bernat’s concept was recently supported by Shapey et al.: in a systematic review on EISOR they did not show any return of spontaneous cerebral or cardiac activity after 5 min of stand-off time [52].

### EISOR

Since the 1960s, vital organ donation has been undertaken in implicit accordance with the dead donor rule (DDR). This stipulates that vital organs must only be removed from deceased donors, and therefore that death must not be caused by the removal of a vital organ. Ever since the introduction of the brain death criteria, the majority of vital organ procurements have been performed on donors who had been declared dead based on these criteria (donation after brain death, DBD). In 1966, the French National Council of the Order of Physicians suggested that organ procurement from donors who were not yet declared dead could be legitimate under certain circumstances. The 2000s saw the introduction of organ donation after circulatory determination of death (DCDD), which was split into two broad categories: controlled (cDCDD), typically preceded by a period of time of critical illness; and uncontrolled (uDCDD), typically succeeding a refractory cardiac arrest [53]. In both cases, the question of when the patient is to be declared dead hinges on the cessation of cerebral perfusion. In the case of organ procurement from ECLS, death would be declared in the limbo between permanent and irreversible [54].

To give an example of a setting in which DCDD may be relevant, a donor would usually present serious cardiovascular and eventually also neurological deficiencies, without fulfilling the brain death criteria. If that individual is neither likely to recover nor eligible for transplantation or VAD, organ procurement for donation would be precluded following DBD and DDR, but not under DCDD, as per Bernat’s concept of the permanence of death. Several protocols for cDCDD and uDCDD have been developed and successfully adopted in Europe and Canada, all of which include a varying no-touch interval of asystole, where all medical support ceases and the organ donor is observed for circulatory auto-resuscitation. However, as Halpern et al. object, the confidence of time needed is based on anecdotal reports or small cohorts [55].

For the same reason, Dalle Ave and Bernat argue that post-mortem interventions like ECLS that could restart circulation to the brain should not be deployed, as this would retrospectively negate the diagnosis. Simultaneous occlusion of the aorta or brain vessels would raise questions about physicians’ complicity in the donor’s death [53, 54, 56].

Halpern et al., as well as Glannon, suggest that DDR should be abandoned [55, 57]. They base this point of view on the fact that the precise transition from permanence to irreversibility in dying is unknown and argue that DDR would invalidate the wish of the donor who has consented to organ donation, as it can jeopardise transplantation. They suggest that the donors’ wish, the absence of suffering and a poor and irreversible prognosis constitute ethically satisfying grounds for the donation of vital organs. By contrast, Wall et al. and Molina et al. hold that uDCDD, performed within the framework of DDR, would be ethically feasible, provided that irreversible death is declared after failed resuscitation attempts [58, 59].

A fascinating prospect for ECLS is the possibility of saving more lives due to a larger population of possible organ donors. However, this can be a double-edged sword, as ECLS could be seen as both an advanced resuscitation tool and an advanced organ-procurement tool [60]. Care should be taken to underline the limitations of ECLS therapy, so that it is not seen as a way of “harvesting” organs from patients who could be resuscitated [61]. The professional roles in transplantation should be strictly separated [53, 62–64].

## Conclusions

In this article, we explored the current state of the debate regarding the ethics of ECLS. In a literature review from the beginning of ECLS to the present, we assessed an extensive corpus of scientific contributions. The complexity of the subject is partly due to rather distinct discourses, dealing with different but interconnected ethical issues over several decades. ECLS itself is complex, however, the discourses seem to be universal.

We therefore suggest that the ethics of ECLS should be recognised as an epistemic concept, in which descriptive facts and ethical norms do not merely supplement but inevitably affect each other. Thus, whether the effectiveness of ECLS is sufficiently supported by evidence, where decision-making authority regarding the limits of ECLS should lie, and whether ECLS should be used in DCDD are all a matter of balancing reasons, rather than deriving norms from facts. Neither dealing with prognostic uncertainty or multilateral communication nor pushing the boundaries of death is novel to the medical profession, but these issues have been particularly aggravated by the evolving technology of ECLS, not least in relation to COVID-19, leaving a great need for careful technology assessment but also individualised practice and open-mindedness from all sides. Few authors may fully subscribe to this, but some are thinking along similar lines.

Early works by Bartlett [3] and Lantos et al. [10] describe how ECLS research is linked to its ethical prerequisites and consequences; Jaramillo et al. [17] point out the rationality of deliberation in limiting ECLS; Carlisle et al. [65] oppose the clear-cut understanding of futility; and in the face of the common utilitarian ECLS-prioritisation model advocated by Abrams et al. [66], Supady et al. [26] prefer the egalitarian approach of Norman Daniels, which is based on the right to participate in a fair and transparent allocation process. Finally, Ross [67] and Halpern et al. [55] depict the notion of death as pragmatic and therefore alterable.

All of these authors contribute in one way or another to the conceptualisation of ECLS as a matter of dialogue. This dialogue, the exchange of arguments and their mutual scrutiny should be seen as the origin of the ethical and factual significance of ECLS and appeal to both legal and healthcare professionals and administrators, patients, surrogates, politicians and the public. It may be justified to represent agents through law, algorithms and expertise, not least when faced with mass critical care needs during a pandemic, but it also signifies avoidance of the argument and could mean that relevant objections remain unheard. Both historical arguments and arguments from deviant discourses can be of great value. For example, the discourse surrounding early neonatal trials may help to improve the future research design in adult critical illness, while the discourse on the ontology and measurability of death may provide valuable knowledge about futility. The present article’s broad overview may help to inform those in charge and those seeking substantiation in the ethics of ECLS.

## Supplementary Information

**Additional file 1.** List of PubMed-cited articles dealing with the ethics of ECLS, categorised by provenance (country), their excerpted ethical content, their corresponding ethical discourse and biographical data.

## Data Availability

Not applicable.

## Abbreviations

ECLS: Extracorporeal life support
COVID-19: Coronavirus disease 2019
ECMO: Extracorporeal membrane oxygenation
V-V: Veno-venous
V-A: Veno-arterial
A-V: Arterio-venous
ECCO_2_R: Extracorporeal carbon dioxide removal
VAD: Ventricular assist device
IABP: Intra-aortic balloon pump
TAH: Total artificial heart
CPB: Cardiopulmonary bypass
EISOR: Extracorporeal interval support for organ retrieval
(E)CPR: (Extracorporeal) cardiopulmonary resuscitation
OHCA: Out-of-hospital cardiac arrest
ARDS: Acute respiratory distress syndrome
DDR: Dead donor rule
DBD: Donation after brain death
(c/u)DCDD: (Controlled/uncontrolled) Donation after circulatory determination of death

## Footnotes

1 ECLS refers to all forms of extracorporeal membrane oxygenation (ECMO) through veno-venous (V-V) or veno-arterial (V-A) cannulation and to extracorporeal CO_2_-removal (ECCO_2_R), through either veno-venous or arterio-venous (A-V) cannulation. Where “ECLS” is not explicitly distinguished from “ECMO”, the terms are understood as synonymous. ECLS does not cover circulatory support without artificial oxygenation, as in ventricular assist devices (VAD), intra-aortic balloon pumps (IABP) or total artificial hearts (TAH), nor does it include the cardiopulmonary bypass (CPB) in cardiac surgery.

2 Basically, the technology of draining and pumping, oxygenating, decarboxylating, filtering and heating or cooling blood before returning it to the patient.

3 Also termed “normothermic regional perfusion”.
